# Equality in sexual health promotion: a systematic review of effective interventions for black and minority ethnic men who have sex with men

**DOI:** 10.1186/s12889-016-3418-x

**Published:** 2016-08-17

**Authors:** Julie Fish, Periklis Papaloukas, Rusi Jaspal, Iain Williamson

**Affiliations:** 1 Centre for LGBTQ Research, Faculty of Health & Life Sciences, De Montfort University, Leicester, LE1 9BH UK; 2 Mary Seacole Research Centre, Faculty of Health & Life Sciences, De Montfort University, Leicester, LE1 9BH UK; 3 School of Applied Social Sciences, Faculty of Health & Life Sciences, De Montfort University, Leicester, LE1 9BH UK

**Keywords:** Black and minority ethnic (BME), Men who have sex with men (MSM), Sexual health and HIV, Evaluation, Implementation research, Systematic review

## Abstract

**Background:**

Over the past decade, new diagnoses of HIV have increased eightfold among men who have sex with men (MSM) of other or of mixed ethnicity in the UK. Yet there is little intervention research on HIV among black and minority ethnic (BME) MSM. This article aimed to identify effective HIV and sexual health prevention strategies for BME MSM.

**Methods:**

We searched three databases PubMed, Scopus and PsychInfo using a combination of search terms: MSM or men who have sex with men and women (MSMW); Black and Minority Ethnic; HIV or sexual health; and evaluation, intervention, program* or implementation. We identified a total of 19 studies to include in the review including those which used randomised control, pre/post-test and cross-sectional design; in addition, we included intervention development studies.

**Results:**

A total of 12 studies reported statistically significant results in at least one of the behavioural outcomes assessed; one study reported significant increases in HIV knowledge and changes in safer sex practices. In 10 studies, reductions were reported in unprotected anal intercourse (UAI), number of sexual partners, or in both of these measures. Six out of the 13 studies reported reductions in UAI; while seven reported reductions in number of sexual partners. Seven were intervention development studies.

**Conclusions:**

Research into the mechanisms and underpinnings of future sexual health interventions is urgently needed in order to reduce HIV and other sexually transmitted infection (STI) among UK BME MSM. The design of interventions should be informed by the members of these groups for whom they are targeted to ensure the cultural and linguistic sensitivity of the tools and approaches generated.

## Background

Men who have sex with men (MSM) continue to be the group most affected by HIV infection [1]. Estimates suggest that 62,880 MSM are living with HIV in the UK, and that an estimated 7,200 MSM living with HIV are unaware of their serostatus. In the general population, approximately 4 men in 1,000 are living with HIV; by contrast, among MSM aged 15–59, 59 men per 1,000 are living with HIV. MSM constituted 55 % of all new HIV diagnoses in 2014 [1]. HIV prevalence is highest in areas of deprivation in England and Wales (E&W), particularly in London. The capital is also the most ethnically diverse area across all of the regions in E&W with above average proportions for most Black and Minority Ethnic (BME) groups [2]. Yet despite recent prioritisation in public health strategies for E&W [3], there is relatively little research from these countries (or the other countries of the United Kingdom) which could underpin effective interventions to reduce sexual risk-taking among BME MSM and promote healthy sexual behaviours. This review is timely given the recognition of the increasing burden of HIV among BME MSM in E&W. It seeks to inform the recent implementation of a strategic framework to reduce sexual health inequalities and ensure that MSM from BME communities enjoy long, healthy lives and maintain fulfilling social and sexual relationships [4]. Because of the organisation of public health agencies in the United Kingdom, the following four sections highlight issues pertaining to the context of E&W.

### HIV diagnoses and transmission among black and minority ethnic MSM

Public Health England collect annual surveillance relating to diagnosis and routes of transmission; these figures indicate that men of white ethnicity comprise 84 % (38,429 of 45,679) of cases of newly diagnosed MSM with HIV in E&W with a route of exposure through sex with men [5]. By comparison, 14.6 % of the total number of men diagnosed (6,654 of 45,679) are among BME men who are exposed in this way. There has also a more than 82 % increase of new HIV diagnoses among ‘Other’ and ‘mixed heritage’ MSM (242 to 442). Increasing proportions of BME MSM who have been diagnosed with HIV have been seen for care: among Black-Caribbean men there is more than 100 % increase (408 in 2005 to 837 in 2014) while among Black African men the increase has been 126 % (267 to 605).

### Defining black and minority ethnic men

Britain has a long-standing history and heritage of different cultures and communities which reflect both its geography and its history. The majority of previous studies around MSM from ethnically diverse groups have been conducted in the USA. While there may be several shared concerns, there are also number of differences between BME communities in E&W and the USA, not only in the terminology used, but also their countries of origin. Minority ethnic groups in the USA constitute 30 % of the population comprising Hispanic (16 %); Black African or American (14 %) with smaller proportions from Asian, mixed, Native American and Pacific Islander groups [6, 7]. In E&W, people from ethnic minority groups constitute 14% of the population [8] with people from the diaspora of India and Pakistan forming the largest minorities.

### The psychological impact of a HIV diagnosis on MSM

The introduction of anti-retroviral therapies (ART) in the mid-1990s has meant that HIV has come to be construed and experienced as a chronic, life-altering, rather than life-limiting illness. Although the majority of HIV-positive individuals with access to ART now have a near normal life expectancy, there is evidence that MSM experience a sense of identity crisis which can be particularly acute in the period immediately following diagnosis [9]. There are a number of psychological sequelae for MSM living with HIV including extreme distress, depression and managing the social stigma associated with the virus. The impact on men’s psychological health may inhibit compliance with public health messages; affect their health-seeking and coping behaviours; their self-efficacy in reducing risk; adherence to medication; quality of life and social well-being [10]. Because no vaccine has been developed for HIV, reducing risk behaviours still constitutes the best strategy for reducing transmission.

Research into BME communities further demonstrates the psychological challenges of HIV infection. Initial responses to a positive diagnosis among members of Caribbean heterosexual communities in London included struggling with multifaceted loss: of their known self, their present life, their envisioned future and the expected role of their partner [11]. Among Black young gay and bisexual men in New York, perceptions of social acceptance were negatively correlated with sexual risk taking [12]. Recognising the impact of a positive diagnosis on psychological health requires that models and interventions for changes in health behaviour take account of distress and the possibility of depression [13]. Public health approaches that aim to increase public willingness to test for HIV, thereby reducing the prevalence of undiagnosed HIV, should be attentive to the psychosocial factors that underpin both testing and a positive diagnosis.

### HIV risks and inequalities

Despite the increasing incidence of HIV among BME MSM, there is relatively little UK research about their distinct risks and health behaviours. Evidence from a three country comparative study of disparities in HIV risks and infection found that only seven per cent of studies were conducted in the UK [14]. There are contradictory findings in existing work on sexual risk-taking among BME MSM. A London clinic study found that BME MSM were significantly more likely to report unprotected anal intercourse with casual male partners in comparison with white MSM [15]. Furthermore, epidemiological research in E&W found evidence that BME MSM are more likely to report high risk sexual behaviour than other MSM, with Black Caribbean and Black African communities in particular experiencing poor sexual health and high rates of bacterial STIs [16]. This study revealed that BME MSM lack culturally appropriate information, safe spaces and social networks to meet their sexual health needs.

By contrast, a meta-analysis revealed that BME MSM were less likely than white MSM to identify as gay men or to disclose their sexuality to others. The study found that BME MSM engaged in fewer risk behaviours, reported less unprotected anal intercourse, had fewer male partners and more condom use during anal sex than other MSM [14]. Yet regardless of the greater likelihood of adopting safer sex behaviours reported in this paper, BME MSM were three times more likely to test HIV positive and six times more likely to have an undiagnosed HIV infection than other MSM [14]. These findings may indicate health inequalities between BME MSM and their white MSM counterparts because early diagnosis and entry into care improve clinical outcomes. The meta-analysis also revealed important differences in HIV risk between MSM in the UK and the USA and underlines the caution required in transferring international research and epidemiological data to a British context:

- BME MSM in the UK are more likely than white MSM to test HIV positive or to ever have an STI or a viral STI;
- BME men in the UK, unlike in Canada and the USA, were more likely to have a history of substance misuse;
- UK BME MSM were more likely to get tested for HIV, but less likely to have heard of post-exposure (PEP) and pre-exposure prophylaxis (PrEP) than were white MSM;
- Among HIV-positive MSM, UK black MSM were less likely to access combination anti-retroviral therapy (ART) than were white MSM;
- In the USA, black MSM engaged in fewer HIV risk behaviours than did other MSM;
- UK BME MSM were equally likely as white MSM to adopt safer sex behaviours;
- BME MSM across the studies were more likely to be affected by structural factors such as unemployment, low levels of educational achievement and having been in prison [14].

Of relevance to this study is the invaluable role provided by community based organisations in supporting people living with HIV. In a study of access to HIV community based services in Northern England, Madden et al. found that attendance was highest in the most deprived areas [17]. Community organisations were shown to provide effective support for the most vulnerable members of society: compared with white UK nationals, attendance was significantly higher among non-UK nationals of uncertain residency status, refugees, migrant workers, temporary visitors and BME groups. The authors suggest the role of community based organisations is vital to the effective management of HIV. Poverty, alongside individual, social and structural factors including migration and HIV stigma and discrimination contribute to sexual health inequalities.

As indicated previously, rates of HIV infection are not evenly distributed across the population; health behaviour theory suggests that the interplay of multiple inequalities compounds HIV risk. Syndemic theory [18] provides an explanatory framework for these co-occurring factors which may include childhood sexual abuse, depression, polydrug misuse [19]; in addition, wider social factors such as migration, poverty and racism may have an ‘additive relationship’ to HIV risk [18]. The current paper builds on a previous review by Maulsby et al. which systematically evaluated the US literature (published prior to 2012) related to HIV behavioural change interventions [20]. This review contextualises MSM behavioural change interventions with reference to HIV and sexual health knowledge and psychological well-being. Since it is intended that this review will inform future interventions in an English and Welsh context, the international literature on efficacy studies was included. In addition, this review includes a more diverse group of BME men and expands the focus to include sexual health interventions in addition to HIV. It also complements a meta-analysis in which Millet et al. [14] found evidence of HIV risks and health inequalities among black MSM in the UK. The review seeks to extrapolate evidence relevant to the E&W context. By answering the question “What constitutes effective intervention research for BME MSM?” we seek to inform the development of implementation research and intervention programmes for these communities in E&W; in addition we aim to underpin public health strategies for this overlooked group of MSM.

### Purpose

- to provide a comprehensive review of the literature on sexual health interventions, in addition to HIV/AIDS;
- to complement an existing review of black (i.e. African-American) MSM by including MSM from additional minority ethnic groups;
- to identify all articles published between 1983–2015. Public health responses for gay male communities were introduced in 1983–4 in E&W [21];
- to identify effective sexual health prevention intervention strategies for BME MSM.

### Aims

- to elucidate shortcomings and gaps associated with existing sexual health interventions. What aspects promote or inhibit attitudinal/behavioural change? What aspects contribute positively to psychological wellbeing?
- to identify effective sexual health prevention strategies for BME MSM;
- to carve out pathways for future research in this area and to provide some preliminary recommendations concerning the development of evidence-based interventions.

## Methods

### Eligibility criteria and study selection

The authors agreed a protocol, which was informed by the updated PRISMA-P checklist for the reporting of systematic reviews [22] following extensive discussion regarding appropriate search terms and relevant databases. We searched three databases PubMed, Scopus and PsycINFO on 16 November 2015 using a combination of search terms: MSM, men who have sex with men and women (MSMW), gay, bisexual, homosexual; BME, black, African, Caribbean, Latin*, Asian; HIV, AIDS, sexual, evaluation, intervention, training, program*, implementation.

We included research articles published in the peer-reviewed literature, as well as on-going and in press studies. Theses, case studies and editorials were excluded. The interventions included were specifically designed for BME/ black/African Americans, Hispanic/Latinos, and Asian and Pacific Islanders MSM/MSMW. Studies solely with MSM participants were selected for inclusion where BME MSM constituted the majority (>85 %) of the sample. Outcomes related to reducing risk behaviours were included such as number of sexual partners, sex with/without condoms; skills, for example, condom use negotiation, as well as psycho-social outcomes such as HIV/AIDS and other STI knowledge, psychological constructs such as self-esteem and social connectedness. Studies conducted in English in countries with black and minority ethnic populations were included. Studies identified by Maulsby et al. were excluded to avoid duplication of findings [20].

### Quality assessment and data extraction

The appraisal of studies was organised in four distinct stages: (1) records identification; (2) records title screening; (3) records abstract screening; (4) full text assessment and final decision for inclusion. Fifty-eight papers were screened by abstract in stage 3 and 28 were retained. We used a modified version of the Critical Appraisal Skills Programme (CASP) tool to evaluate papers for methodological rigour and data relevance (Retrieved 20.11.15 from http://www.casp-uk.net). Consisting of 9 criteria, each item was ranked on a three-point scale (0 = weak; 1 = moderate; 2 = strong) and were appraised for eligibility and inclusion (see supplementary data). Quality assessment was undertaken by two reviewers and decisions made through discussion, involving a third reviewer as necessary. On consensus, each study was scored a quality rating: studies scoring 13–18 were scored as ‘high’ quality; 7–12 were ranked as ‘moderate’ quality; and 1–6 scored a ‘low’ quality rating. The quality appraisal ratings for each included study are presented in the supplementary data file. In stage 4, data from each retained paper were entered into extraction tables giving details about participants, interventions, comparators and outcomes (PICO) [23]. Behavioural outcomes include: number of sexual partners, sex with condoms, oral sex; and the review also identified psychosocial outcomes: for example, negotiation of safer sex and self-esteem by which men develop skills to protect their own health. A PRISMA flow chart identified the results at each of the four stages (see supplementary data) in accordance with PRISMA methodological guidance [22]. We conducted an integrative review of the data from the 13 efficacy studies which allows for the inclusion of experimental and non-experimental research which is the most appropriate due to the heterogeneity of the studies [24].

### Data analysis and synthesis

The results were analysed and synthesised drawing on an approach similar to that proposed by Whittemore and Knafl [24] of data reduction, data display, data comparison and verification of conclusions. The data were reduced by extracting key findings which were then displayed (Table 1). This enabled an iterative process of identifying patterns and themes. Results were then grouped together into two overarching categories of behavioural and psychosocial outcomes. At the presentation stage, explicit details from the primary sources were included to support the conclusions drawn.

**Table 1:** Behavioural and pyschosocial interventions

First author	N	Name	Location	Theory	Description	Design	Primary Outcome	Secondary Outcomes	Findings	Limitations
Adam (2011) Quality Appraisal score: 11	40 Spanish speaking gay, bisexual and other MSM	Mano en Mano Latino	Toronto, Canada	NA	An initial day-long session, followed by four two-hour evening sessions. A life-skills and HIV prevention program.	Pre and Post-test	Frequency of protected and unprotected anal intercourse	Loneliness. Self-perceived degree of social connection/self-isolation	Decrease of UAI from pre-test to post-test stat sig. (*p* = 0.004). No difference between scores in loneliness.	Small sample size. No control or experimental group. Self-report (social desirability bias). Different groups thus not a coherent single group.
Carballo-Dieguez (2005) Quality Appraisal score: 13	180 Latinos or of Latin American descent	Latinos Empowering Ourselves (LEO) LGBM (Latin gay and bisexual men)	New York City	Freire’s Theory; Empowerment Ideas; researchers’ own quantitative and qualitative research	Eight two-hour group sessions, once a week. Basic exercises and focusing on a specific theme. Session themes included oppression, transgression of rules, excuses, substance use, goal setting, the role of pleasure, self-efficacy, and plans for the future.	Randomised Control Trial (RCT)	Unprotected Anal Intercourse (UAI)		46 % of the intervention participants from baseline to follow-up 1 reported no UAI. For the control group this was 54 %. 44 % of the intervention participants at follow-up 2 from intervention reported no UAI. 40 % from control. No differences between groups.	Selection bias: Participants ready to change
										*Assessment effect*: Elicitation of critical consciousness comprehensive baseline assessment.
										Convenience sample. Self-report – social desirability biases.
Choi (1996) Quality Appraisal score: 17	329 Asian and Pacific Islanders (API)	NA	San Fransisco, USA	Health Belief Model; Theory of Reasoned Action; Social Cognitive Theory	A single three-hour group session.	Randomised control trial (RCT)	Number of sexual partners	UAI and AIDS-related knowledge, attitudes and skills	Statistically significant Reductions in number of sexual partners at 3 month follow-up (p = 0.0004)	Convenience sample Self-report, Social desirability biases
					Increasing Positive ethnic and sexual identity; acknowledging HIV risk behaviours; presenting positive images of H-API. Enhance AIDS knowledge, attitudes to safer sex and sexual negotiations skills.					
Harawa (2013) Quality Appraisal score: 15	437 bisexual black men	Men of African American Legacy Empowerment Self (MAALES)	Los Angeles, USA	Theory of Reasoned Action and Planned Behaviour; Empowerment theory; Critical Thinking and Cultural Affirmation model	Six two-hour small-group sessions conducted over three-weeks plus booster sessions at six and 18 weeks post intervention. Holistic behavioural change	Randomised Control trial (RCT)	Number of male/female/male to female trans intercourse partners; number of any anal and vaginal intercourse; any unprotected intercourse (UI); any unprotected serodiscordant intercourse; sex while using substances		Intervention: Reduction in UAI with males (*p* = .04); reduction in UAI/UVI with females (p = .01); UI with male or females (*p* < .01); reduction in number of partners both male and female (p = .01); any risky drug use borderline significant (p = .04). No significant differences in sex while using substances.	Convenience sample The focus is mostly on female partners. Self-report and Social desirability biases.
Hosek (2015) Quality Appraisal score: 13	456 Black young men who have sex with men (YBMSM) and 50 young black persons as opinion leaders	Promoting Ovahness through Safer Sex Education (POSSE)	Chicago, USA	Popular opinion leader (OL) model	Opinion leaders trained during four 2 h sessions at risk-reduction conversation. Then, out in the community (House Ball Community) to spread knowledge among their peers in relation to sexual health knowledge	Repeated cross-section surveys (five separate circles)	Number of sex partners; Number of condomless anal intercourse (CAI) acts; any CAI with male partners; Sex with partner of unknown serostatus; any oral or anal act with unknown serostatus partners	Frequency of sex under the influence of substances	Decline in mean number of male sex partners (*p* = 0.004); Statistically significant difference in oral or anal sex with unknown status male partners (p < 0.001); no statistically significant differences in all other outcomes.	No control group for this study. Convenience sample which might cause generalisability issues. Self-report bias
Jemmott (2014) Quality Appraisal score: 16	503 African American MSM	Being responsible for Ourselves (BRO)	Philadelphia, USA	Social Cognitive Theory; the reasoned-action approach; qualitative research (formative research with black MSM).	A three session one-on-one HIV/STI risk reduction designed to increase consistent condom use vs. three one-on-one health promotion intervention.	Randomised Controlled Trial (RCT) – cross-sectional study.	Condom-protected intercourse acts	Proportion of condom-protected intercourse acts; unprotected sexual intercourse; multiple sexual partners; IAI; RAI. Theoretical constructs: condom use hedonistic outcome expenctancy; condom use prevention outcome expectancy; condom use self-evaluative outcome expectancy; condom use availability self-efficacy; condom use negotiation self-efficacy; condom use technical skills efficacy; condom use impulse control self-efficacy; HIV risk-reduction knowledge; condom use knowledge.	Increase in consistent condom use at 90 days post intervention follow-up (*p* < 0.0001, CI 95 %, 0.73-1.45); proportion condom-protected intercourse (p < 0.0001, CI 95 %, 0.87-2.77); unprotected intercourse (p < 0.0001, CI 95 % 0.68-1.35);multiple sexual partners (p < 0.0001, CI 95 %, 0.80-1.43); insertive anal intercourse (p < 0.0001, CI 95 %, 0.81-1.51); receptive anal intercourse (p < 0.0001, CI 95 %, 0.57-1.13). There were no differences between the arms of the intervention.	One-on-one approach instead of group intervention which might have affected the results. Black populations more socially close-knit; an aspect important in relationships related negotiations. Self-report/social desirability bias. Generalisability limited as participants not randomly selected.
					Baseline, IPT, 6-months follow-up; 12-months follow-up.					
O’Donnell (2014) Quality Appraisal score: 15	346 Latino MSM	No excuses/Sin buscar excusas	New York, USA	Social Cognitive Theory	Single 45–60 min session intervention (either English or Spanish) vs. non-attention control condition. Three-month follow-up	Randomised control trial/Randomised field trial	1) total number of unprotected anal intercourse (UAI) acts with last two male partners; 2) condom use at last intercourse with a male partner; 3) self-report of an HIV test during the 3-month follow up window.		Decline in mean number of UAI (59 % vs. 39 %, F = 4.10, *P* < 0.05); Intervention participants more likely to engage in condom use after intervention (AOR = 1.69; 95 % CI 1.02-2.81, p < 0.05).	Short-term impacts (3-month follow-up). Self-report bias. Convenience sample.
Stein (2015) Quality Appraisal score: 14	337 young MSM of colour (Black/African-American and Latino/Hispanic males).	Community based organisation behavioural (CBO) outcomes of Many Men, Many Voices (CBOP-3MV) Project	New York, Tampa, New Orleans, USA		Small group-level intervention facilitated by peers in groups of 6–12 clients.	Repeated measures design with no control group	1) Sex partners: Number and prevalence of sex partners; number of serodiscordant/unknown status partners;		Significant decreases in all outcomes, both Sex partners and sex events related.	No control group for the specific study. Internal validity is not strong. Self-report bias. The three different CBOs offered the intervention in slightly different ways.
							2) Sex events: Number and prevalence of sex events without a condom; number of sex events with serodiscordant/unknown status partners; number and prevalence of sex events without a condom with another (male); number of sex events without a condom under the influence of substances.			
Somerville (2006) Quality Appraisal score: 13	766 Young Latino MSM and 37 Young Latino Promotores (YLP)	Young Latino Promotores (YLP)	Vista, California; McAllen, Texas, USA	Popular Opinion Leader model – Theory of diffusion	Uses social networks to deliver HIV prevention messages. Identification and enlistment of popular persons within the community. Training them in disseminating prevention and risk reduction messages related to HIV/STIs. Supporting and reinforcing successful waves of POL in order to create a culture of normative change.	Repeated cross-section surveys	1) HIV Risk behaviour: frequency of receptive anal sex with condoms; frequency of receiving and giving oral sex without using condoms;		Most of the items observed were already high. Increase in the use of condoms in receptive anal sex (*F* = 5.797; *p* < .01), also increase in frequency of giving oral sex (*F* = 3.928; *p* < .01). They report increase in HIV/AIDS knowledge in Awareness item “sharing clothes and hats not a mode of transmission” (F = 6.671; p < .01). Finally, they report statistically significant differences in acceptance of safer sex (F = 4.811; p < .05).	No control group which impacts integral validity. Self-report bias and social desirability biases. Convenience sample. Cross section surveys with different participants each time and not the same participants.
							3) HIV/AIDS knowledge 2) Social norms: acceptance of safer sex;			
										The theory of diffusion which OL is based recommends 15 % of the population should be trained as OL in order for change to occur. This is not the case for this particular study.
Tobin (2013) Quality Appraisal score: 16	147 African American MSM	Unity in diversity (UND)	Baltimore, USA	Information-motivation-behaviour model (IBM); social network theory (SNT); social cognitive theory (SCT) – self-efficacy construct.	Six group sessions group sessions as the intervention arm and a single session as control condition. 3-month follow-up	Randomised control trial (RCT)	Number of partners; condom use; sex while drunk or high.		Increased odds of reporting fewer male partners (AOR = 3.03; 95 % CI = 1.26 – 7.28); marginal effects on condom use with male partners and with partners with HIV-negative/unknown serostatus (AOR = 2.64; 95 % CI = 0.95-7.36) (AOR = 3.19; 95 % CI = 0.98-10.4).	Short term results. Convenience sample which might impact generalisability. Self-report and social desirability biases. Potential contamination between conditions.
Vega (2011) Quality Appraisal score: 13	113 Latino gay men	SOMOS: We are	New York, USA	Social Identity Theory	Five group sessions exploring concepts of sexual, ethnic and cultural identity; coming out process; body image and sexual acts. Ten intervention cycles.	Pre- and post test	HIV/AIDS and hepatitis C knowledge; number of sexual partners, different types of partners, high risk sexual encounters; psychosocial constructs (coping, self-efficacy, internalized homophobia, self-esteem, sources for social support and collective self-esteem).		Increase in HIV/AIDS and hepatitis C knowledge from baseline to 90 days follow-up (*t* = 10.84, *p* < 0.05), hepatitis C knowledge (*t* =12.87, *p* < 0.05); number of sexual partners decreased (t (112) = 4.33, *p* = 0.000); decrease in high risk sexual behaviours in 180 days follow-up (*t* = 4.76, *p* = 0.000); increase in specific psychosocial constructs: Self-esteem, coping, Social provisions (all *p* < 0.05) and Collective self-esteem items (Identity; Public; public Latino: all *p* < 0.05)	No control group which might increase the self-report bias and create problems of integral validity. Self-report and self-desirability biases. Sample size not randomised but rather specifically focused on Latino populations in New York – issues of generalisability
					Baseline, Follow-up 90 days, follow-up 180 days					
Williams (2013) Quality Appraisal score: 15	88 HIV+ African American MSMW with history of childhood sexual abuse.	Enhanced Sexual health Intervention for Men (ES-HIM)	Los Angeles, USA	Cognitive-behavioural approach and ecological framework	Six two-hour small group sessions administered over a period of three weeks. A stress-focused sexual risk reduction intervention condition (ES-HIM) vs. a general health promotion intervention condition (HP).	Randomised control trial (RCT)	Sexual risk behaviours (unprotected anal and vaginal sex); number of sexual partners; psychological symptoms; stress biomarkers (urinary cortisol and catecholamines and neopterin (indicator of HIV progression).		Both groups reduced unprotected anal insertive sex (time *P* < .01). Both reduced vaginal sex (time *P* < .01). Both groups reduced number of sexual partners which was sustained at the 6-months follow-up as well as time *P* < .001). Reductions in depression and PTSD (time *P* < .01). Overall, intervention arm showed no significant advantage over control group.	Participants despite randomisation differences at baseline in CSA severity, psychological symptoms and biomarkers. Major intervention group effect (ES-HIM lower biomarker composite scores than HP at baseline). Failure in randomisation that could not be corrected due to small sample size. Convenience sample which might impact generalisability. Self-report and social desirability biases.
					Assessment baseline, 3–6 month follow-ups.					
Young (2013) Quality Appraisal score: 13	112 African American and Latino MSM	HOPE (Harnessing Online Peer Education) UCLA	Los Angeles, USA	The community-peer leader model. Similar to the opinion leader model.	Twelve-week HIV prevention intervention designed to use peer-led Facebook groups to diffuse HIV information to increase testing among black and Latino MSM. Home-based HIV testing kit and completed questionnaire at baseline and 12-week follow-up. Intervention: received peer-delivered information on HIV prevention. Control: Peer-delivered information on general health information	A hybrid design of RCT with diffusion approach	Behaviour change: requesting a home-based kit, returning the kit and following-up for test results	Number of sexual partners, observed and self-reported communication using the social networking community.	More intervention participants requested and followed-up with a kit (25–8 vs. 11–0); high participation and engagement rates in both groups; number of sexual partners decreased in both groups.	The statistical data are not present for the primary outcome as the numbers are low, small sample.
										Self-report bias. Convenience sample.

## Results

Our research identified 173 records after duplicates were removed. Of these, 115 were excluded after two members of the team screened them independently. Fifty eight were retained for screening by abstract. A total of 28 studies were deemed to be potentially relevant for this review and included for full-text assessment. Of these, nine were excluded: six were not intervention studies, two did not focus on BME MSM and one was conducted in a country with a Latino majority population. A total of 19 studies were included in this review of which 13 had findings published in peer-reviewed journals, six were peer-reviewed studies at an intervention development stage, and a recently completed ‘grey’ study in E&W [25]. We were unable to identify any published studies of HIV or sexual health interventions among BME MSM in E&W. Table 1 gives a brief description of the citation, country of origin, aim of the study, theoretical orientation, participant characteristics, methods and comparators, primary and secondary outcomes; limitations and conclusions.

Study ***participants*** were of African-American, Latino (Spanish speaking) and Asian Pacific Islander heritage. Most studies were US based and participants were drawn from the predominantly urban communities of New York, San Francisco, Los Angeles, Toronto, Chicago and Baltimore. Some studies were targeted to particular sub-populations of MSM communities including the African-American House Ball community, Mexican farmworkers, young MSM of colour, behaviourally bisexual men, injection drug-using MSM and BME men who had experienced sexual abuse. Participants were men with HIV negative, positive and unknown status. Typically, men were recruited through convenience sampling, assessed for eligibility by screening interview and randomly assigned to an intervention or a control group. Study sample sizes ranged from 40–503 participants.

Studies adopted diverse ***theoretical perspectives*** and ***domains of interest*** which ranged from psycho-social concerns, HIV testing to the assessment of cortisol levels in urine samples. Themes included men’s social context: social isolation, migration, stigma and oppression, developing a positive identity, body image, social support; HIV prevention, risk reduction and condom use; lifestyle concerns: diet, smoking and exercise; satisfying sexual behaviour.

Six studies were conducted among Latino/ Spanish speaking populations, seven studies were undertaken among Black/ African-American communities, one of Asian Pacific Islander and two studies included both Latino and Black/African American men. Strategies adopted to ensure the ***cultural sensitivity of interventions*** included developing collaborative partnerships with a range of Community-Based Organisations such as health centres catering for gay and bisexual men’s communities alongside organisations for particular ethnic or cultural groups (e.g. the Centre for Spanish Speaking Peoples). In some studies, Spanish constituted the working language of the research team and the advisory committee meetings and interventions were all conducted in Spanish. Methods included the use of culturally appropriate materials such as a commissioned video, sexual diaries, word association, problem solving, analysis of Spanish proverbs, surveys, interviews and focus groups. Five studies were informed by the work of Diaz, some of them adapted or used the programme he developed in the handbook Hermanos de Luna Y Sol.

### Intervention design

Seven out of the 13 efficacy studies [26–32] used a ***randomised controlled trial (RCT)*** design in which participants were randomly assigned to the experimental or the control condition. Of these seven studies, two [26, 27] had an experimental condition and a waiting list as the control condition. In two studies [30, 31] the control condition comprised of general health promotion focusing on diet and exercise; for two studies [28, 32] the control condition consisted of a HIV risk reduction session (e.g. based on a standard HIV test counselling approach of 15–25 min); and in one, the control group were offered a HIV test [29]. The length and number of sessions varied across the studies: from a single 45–60 min long intervention [29] to an intervention with twice weekly 2-h sessions over a three week period [24]. Two studies in this review used a ***pre-post design*** [33, 34]; two studies used a ***repeated cross-sectional design*** [35, 36]; one used a ***mixed design*** of RCT with a repeated cross-sectional design [37], and one used a repeated measures design with no control group [38].

The studies revealed innovative and diverse ***approaches to HIV prevention intervention***. Seven studies used group-based approaches; one study used individual sessions while another used a combination of group–based and individual sessions. Three studies adopted the Popular Opinion Leader intervention, modelled on the work of Kelly [39], which provides training for peer leaders to enable them to use social networks to deliver HIV prevention messages. This approach was used in particular to deliver risk reduction messages to ‘clandestine’ or marginalised groups such as Mexican farmworkers and the House Ball community.

### Measures for assessment

In eight out of the 13 efficacy studies, the primary outcomes assessed were: unprotected anal intercourse (UAI), unprotected anal and vaginal intercourse (UAVI) and condom protected intercourse (CPI). Six of the 13 studies used the risk behaviour outcome measure of reductions in the number of sexual partners between pre and post intervention. Secondary outcome measures included reductions in sex under the influence of substances (2), increased HIV testing (2), HIV/AIDS knowledge and HIV risk-behaviour knowledge (1) and psychological and social constructs of human behaviour (3). One study (Williams) assessed UAVI alongside bio-physical markers to ascertain stress levels.

### Reductions in behavioural risks

A total of 12 studies reported statistically significant results in at least one of the behavioural outcomes assessed [26–35, 37, 38], while Somerville [36] reported significant changes in safer sex practices. Across ten out of the 13 efficacy studies, reductions were reported in unprotected anal intercourse (UAI), number of sexual partners, or in both of these measures [28, 30, 37]. Six out of the 13 studies reported reductions in UAI [27, 30, 32–34, 37]. Seven studies reported reductions in number of sexual partners [28–30, 32, 35, 38, 39]. These sexual behaviours are considered to increase the risk of HIV/ other STI transmission. In three studies, the reductions in UAI and in the number of sexual partners occurred in both the efficacy arm of the intervention and in the control group [27, 31, 32].

The sole individual intervention [30] included in this review found significant *overall* declines in UAI, but there were no significant differences between the experimental and the control conditions. Two studies adapted the Popular Opinion Leader model: *Promoting Ovahness through Safer Sex Education* (*POSSE*) [35] and the Young Latino *Promotores* project which also aimed to build capacity in community based organisations to tackle HIV prevention [36]. Statistically significant declines were observed for multiple sexual partners, UAI with any male partners, and with male partners of unknown HIV status [36]. Increase in safer sex using condoms was observed, but this was not statistically significant [36].

Four RCT studies [27–30] showed behavioural change in the intervention group in comparison with the control condition. Two RCTs showed significant results across the overall sample [26, 31]. Two studies which showed low to moderate effectiveness used the Popular Opinion Leader model with a pre-post design [35, 36]. A pre-post study by Vega reported significant reductions in number of sexual partners and in high risk sexual behaviours (i.e. UAI) [34].

### Psychosocial outcomes

Increases in knowledge about HIV/AIDS were observed in three studies [31, 34, 36]. Vega [34] showed increases in psychosocial constructs of self-esteem, coping, social provisions and collective self-esteem (that is, public Latino identities). One study found significant increases in knowledge together with increased social norms about the acceptance of safer sex [36]. A study of men who had been sexually abused in childhood found reductions in depression [31].

We have also identified six ***intervention development studies*** (Table 2) [40–45], of which *I am men’s health* [40] is the only study where PrEP adherence formed the outcome measure. In this study, an overall compliance rate of 73 % among young black MSM was found. This is important as PrEP is an emerging HIV prevention tool that has gained ground among groups at high risk of HIV acquisition, such as MSM in San Francisco (although at the time of writing PrEP is not licensed for use in the UK). A formative study using focus groups [41] sought to inform the development of a mobile phone-based HIV intervention; findings suggested the need for a smartphone application or website with a text messaging component. A second feasibility study using focus groups (*N* = 105) explored the use of web-based HIV prevention for drug injecting Black MSMW [42]. Findings suggest the need for dedicated space with HIV prevention programmes for this group of men which should include holistic services including job assistance. Solorio developed HIV prevention messages for young Latino MSM who do not identify as gay and translated and tested these messages as Public Service Announcements [43]. HealthMpowerment.org is a mobile phone-optimised online intervention with young BME MSM and transgender women which reported statistically significant improvements in social support, social isolation and depressive symptoms [44]. HOLA en Grupos is a Centre for Disease Control supported evaluation of behavioural interventions for potential use with Latino communities [45].

**Table 2:** Intervention Development Studies

First Author	N	Name	Location	Theory	Description	Design	Primary Outcome	Findings	Limitations	Future Steps
Daughtridge (2011)	23 young MSM of colour	I am Men’s Health	Philadelphia, USA	Intervention piloting of PrEP with yMSM of colour. Drug adherence.	The participants are offered an initial screening process where HIV status is ascertained and if they are HIV-negative they are offered the first 7 pills of Truvada (PrEP treatment). Time on PrEP ranged from 1 – 28 weeks	Adherence intervention. Measurement of tablets collection. Workshops offered in educating the participants on safer sex, alongside PrEP	Adherence to PrEP treatment.	Following the specific methodology the retention rates are quite high. Consistence adherence over time. Overall adherence 73 %. Ongoing, the adherence for the first 4 weeks reaches 80 %	Cost, however the effects can be cost significant if PrEP offered to at-risk populations (e.g. yMSMc). Adherence only illustrates indirect HIV prevention success. There is a need for further research (i.e. RCT design).	Implement a more accurate measure of adherence Scale the programme up.
Hightow-Weidman (2015)	15 young black MSM and transgender women (YBMSM/TW)		North Carolina, USA	Pilot study. Online intervention based on HealthMEmpowerment.org (HME) online mobile-optimised intervention	This is the next phase of the Muessig (2013) IDS project.	One month pilot online intervention study. Measuring pre and post intervention acceptability. Outcomes included psychosocial constructs and outcomes associated with sexual risk behaviours.	Sexual risk behaviour related outcomes and psycho-social constructs.	There were statistically significant improvements in social support (*p* = .012), social isolation (*p* = .050), and depressive symptoms (*p* = .045)	No control group. Small sample size. The length is too short (one month) as this is a pilot study.	Implementing the final stage of this intervention development in the form of a large scale RCT.
Muessig (2013)	22 young black MSM	NA	North Carolina, USA	Formative research in electronic and mobile based intervention.	Informing the development of a mobile phone-based HIV intervention. Second round of formative research. Focus groups discussion. In the focus groups, the primary set of discussions explored daily use and feature preferences of mobile phones, websites, and applications. Daily e-journal responses. The participants worked as experts in informing the content of the intervention	Focus group discussion, demographic survey and e-journals.	NA	Importance of communicating by mobile phone texting. There is a need for enhancing a mobile phone application or website with a text messaging component. Also, the participants were interested in information in general sexual health issues.	Not representing the population. Small number of focus groups.	The incorporation of interactive application features for the HealthEmpowerment.org intervention having in mind the specific needs of this particular group of people. Promising opportunities for health interventions via mobile and web oriented technologies. However, they recommend avoiding one-size-fits-all health messages, as there is a need for acknowledging cultural and individual sensitivity.
Rhodes (2015)	Latino/Hispanic MSM	HOLA en Groupos	North Carolina, USA		A description of the enhancement of HOLA en Grupos, a community-based behavioural intervention for Latino/Hispanic MSM which is currently implemented	The convergence of representatives from different institutions working with Latino/Hispanic MSM in order to exchange best practice in order to enhance the HOLA en Grupos intervention.		This enhancement project maintains the original format of the Hola en Grupos intervention; it incorporates the experiences of Latino MSM as well as the input of service providers.		Enhance the HOLA en Grupos. Consequently, if HOLA en Grupos is effective it will be implemented on a larger scale.
Solorio (2014)	61 young Latino Immigrant MSM		Seattle, USA	Integrated Model of Behaviour	Application of data from this formative research to develop HIV prevention messages in order to promote timely HIV testing and then to translate these into public service announcements (PSAs) through a marketing informed technique. Initial step in the development of theoretically based HIV prevention messages for Latino MSM that may be used in campaigns for HIV prevention.	Intervention Development	A number of different steps and stages	Development of HIV prevention messages through previously collected formative data; testing PSAs within the Latino MSM community; recognition of important factors to consider when developing HIV prevention messages. Focus group discussion with Latino MSM.	Issue of generalisability as the participants where monolingual Spanish of only Mexican descent; recently immigrated to the US.	Launching a campaign with which Latino MSM will be helped to receive access to confidential HIV testing and information. The creation of a culturally tailored HIV awareness campaign that the target group could relate to. Delivering the programme through selected channels (e.g. mass media).
										Forthcoming evaluation of the media campaign.
										Recommendations for studies to use more rigorous RCT designs.
Washington (2010)	105 Sex-Trading Injection Drug–Using Black MSMW		Baltimore,USA		Information and material relevant for the HIV prevention needs of Injecting-Drugs Users Men who have Sex with Men and Women. Issues which need addressing in order to motivate safe sex practices.	Intervention Development – Exploratory study	Qualitative approach: Focus groups. Self-report demographic study as well.	Findings: 1) Need for safe place for information-seeking which is tailored to their specific needs. 2) Prevention programmes with comprehensive services. 3) Communication skills/prompts for negotiating safe sex.		

## Discussion

The overall results of this review indicate ***moderate to high efficacy*** of behavioural change interventions in African-American, Latino and Asian and Pacific Islander (API) men exclusively in the North American context. Six studies showed reductions in condomless anal intercourse, while seven studies showed a decline in the number of sexual partners: in the absence of other prevention methods, these behaviours place men at increased risk of HIV acquisition.

A number of the studies reported extensive preparatory work to ensure the relevance of interventions, including the development of culturally sensitive approaches and materials. Many studies adopted a holistic approach exploring the context for men’s sexual behaviours within the realities of their everyday lives, which is also highlighted in psychotherapeutic approaches to HIV prevention [46]. Moreover, consistent with previous research into the potential psychosocial antecedents of sexual risk-taking, interventions [47, 48] addressed social issues including housing and migration, psycho-social constructs such as isolation, self-esteem and negotiating skills, inter-personal concerns, for example, building social networks in addition to men’s access to health and social services. Fewer studies in the review showed evidence of psycho-social change following the intervention, with the exception of SOMOS which reported increases in psychosocial constructs e.g. self-esteem and coping while Es-Him found a reduction in depression [31]. Social isolation continues to be a predictor of sexual risk-taking and is associated with other factors which increase risk such as depression and substance misuse [33].

More specifically, these psychosocial factors may reduce the individual’s engagement with ability to negotiate safer sex practices, such as negotiating condom use, discussing HIV with partners, or adhering to pre-exposure prophylaxis (PrEP). Previous research has indicated that behavioural change interventions alone will not lead to reductions in HIV or sexually transmitted infections among BME MSM because recent increases in diagnoses do not seem to be attributable to an increased prevalence of at risk behaviours in comparison to other MSM [14]. Rather, it is necessary to increase knowledge regarding transmission of HIV and other sexually transmitted infections and willingness to undergo appropriate testing [49, 50]. Knowledge of HIV prevention methods, other than the use of condoms, such as PEP and PrEP is low among BME MSM, which can contribute to increasing HIV incidence [51]. Moreover, they are less likely to access care and continue using care and treatment regimens in comparison with other MSM. This can further exacerbate health inequalities among BME MSM [52]. In a UK study of 16,406 BME MSM, the proportion with no follow up after HIV diagnosis was higher than among white MSM. Permanent loss to follow up was highest in other/mixed groups and lowest in Indian/Pakistani /Bangladeshi groups. The importance of follow up is underscored because once BME MSM are receiving ART, there are no differences in virological, immunological and clinical outcomes [53]. These findings highlight a need to extend culturally sensitive approaches to the wider healthcare environment.

A number of factors contributed to the effectiveness of the interventions in our review in reducing HIV risk behaviours. Notably, studies were underpinned by theoretical frameworks and risk behaviours were addressed in the wider context of men’s lives. Thus, it appears that those interventions that integrate community-based knowledge in broader theoretical frameworks regarding risk-taking behaviour are more likely to be effective. Interventions formed an integrated programme working alongside community based organisations, some were concurrently conducted in multiple cities, studies were conducted over a 3 – 6 month timescale following the intervention, retention rates were high and studies used a range of incentives to minimise attrition. This suggests that future interventions can be conducted and evaluated over longer time periods during which actual behaviour change is most likely to be observable.

### Intervention development studies (IDS)

A number of pilot studies and intervention development research projects were also recognised. There is a promising trend towards designing behavioural and psychosocial change approaches through a complex, iterative and multiple staged process involving individuals who are members of the communities for which the interventions are designed. This can help facilitate a culturally and linguistically appropriate perspective that takes into consideration the factors that are likely to promote positive engagement with the intervention. This cultural perspective is then integrated into theoretical models of behaviour change. Some of the IDS were focused on raising health awareness, providing HIV/STIs prevention or offering sexual health training through electronic means of communication. These IDS were pilot interventions planned and disseminated through contemporary methods of communication (e.g. social media and mobile communication). This is consistent with the prominence of internet, mobile and application communication within young MSM communities with regard to gay in-group socialising, and sexual partner seeking [54]. Finally, we identified one study [40] that discussed adherence to pre-exposure prophylaxis (PrEP) in young MSM of colour. This is important in view of the promising findings from recent clinical trials that PrEP constitutes an effective barrier against HIV infection among MSM [55]. Moving beyond the use of condoms as the sole HIV prevention strategy, this emerging work on PrEP could further complement and enhance existing predominantly condom-based HIV prevention interventions among BME MSM communities.

### Limitations

While this systematic review identifies a number of important factors that underpin effective sexual health interventions, there are some limitations. Specifically, the review focussed on papers published in peer reviewed journals and excluded grey literature. Although the grey literature may not be methodologically robust, it might indicate specific insights which may lead to new research directions. This focus may have biased the results of this review towards studies with significant findings and may have excluded studies with null findings. Moreover, the existing evidence base suffers from some methodological limitations, such as the existence of conflicting and limited evidence which can preclude the formation of robust interventions. For instance, there are inconsistent findings about the link between PrEP use and condom use. While PrEP is protective against HIV, its use without condoms could place MSM at risk of other STIs. The heterogeneity of populations included in studies may also be problematic given the distinct cultural and linguistic needs of particular BME MSM communities. Many of the interventions include only limited follow-up periods which can make it difficult to assess the robustness and duration of the intended behavioural changes. The changing nature of HIV risk-related behaviours and the diverse prevention methods employed by MSM, such as serosorting, strategic positioning and biomedical prevention methods, are not currently reflected and represented in the current interventions and as yet, evaluation of these strategies are poorly represented in the extant literature. Other limitations include the heterogeneity of intervention content and of the outcome measures. Despite these limitations, the review makes an important contribution to developing the knowledge base to inform future behavioural and psychological interventions for BME MSM. The review provides strong evidence that effective interventions for this previously overlooked group of men are underpinned by relevant theoretical frameworks, cultural sensitivity and the involvement of potential users in both the design and delivery of health education and behavioural change approaches. The limitations acknowledged here should be taken into consideration in the development of future interventions.

## Conclusions

Despite the relevance of HIV and sexual health risk prevention for BME MSM, this review has not produced any intervention studies conducted within a UK context. Research into the mechanisms and underpinnings of future sexual health interventions is urgently needed in order to reduce HIV and other STI infection among UK BME MSM, who remain a high risk group [3]. There has understandably been a focus on condom-based approaches to HIV prevention but as the contemporary prevention landscape develops, additional approaches will need to be considered. These include biomedical prevention options, such as PEP and PrEP, and seroadapative behaviours, such as serosorting, strategic positioning in sexual encounters, and modification of sexual behavioural practices. In short, while condom use constitutes a highly effective HIV prevention option, there are other options that should also be considered when evaluating future interventions. HIV prevention agencies are increasingly recognising the need to promote other safer sex strategies in addition to condom use. In order for future interventions to be successful, there is an imperative need to acknowledge not only the cultural and linguistic specificities of the groups targeted but also the role of stigma and medical mistrust [56]. While materials developed for North American studies with Hispanic Latino communities for example, may not be relevant to the UK BME communities (i.e. Brazilian/Portuguese speaking communities, South Asian communities, African-Caribbean communities), this review has highlighted methods which may be applied in the British context. For example, effective interventions are characterised by an integrated approach which recognises the complex interplay between behaviours, knowledge and psychosocial factors, such as skills in negotiating safer sexual practices. Health and environmental communication models also suggest that approaches that take identity into account are more likely to be effective in promoting public understanding and behaviour change [57]. The inclusion of “insider” perspectives in the design of appropriate interventions, i.e. from members of the group being targeted, is advantageous because it may facilitate a culturally and linguistically sensitive approach, and also reduce feelings of suspicion and outgroup threat that have been observed in some attempts to reduce sexual risk-taking [44]. The response to such interventions will conceivably be more favourable. Finally, the review indicates that interventions should be structured over a timeframe that allows for the collection and analysis of follow-up data, particularly as behaviour change can take a while to manifest itself in evaluation research.

## Abbreviations

ART, anti-retroviral therapies; BME, black and minority ethnic; CPI, condom protected intercourse; IDS, intervention development studies; MSM, men who have sex with men; MSMW, men who have sex with men and women; PEP, Post-Exposure Prophylaxis (PEP); PICO, participants, interventions, comparators and outcomes; PREP, pre-exposure prophylaxis; RCT, randomised control trial; STI, sexually transmitted infection; UAI, unprotected anal intercourse; UAVI, unprotected anal and vaginal intercourse
